# Radar–Camera Fusion in Perspective View and Bird’s Eye View for 3D Object Detection

**DOI:** 10.3390/s25196106

**Published:** 2025-10-03

**Authors:** Yuhao Xiao, Xiaoqing Chen, Yingkai Wang, Zhongliang Fu

**Affiliations:** 1 Chengdu Institute of Computer Application, Chinese Academy of Sciences, Chengdu 610213, China; 2 The School of Computer Science and Technology, University of Chinese Academic of Sciences, Beijing 101408, China

**Keywords:** 3D object detection, autonomous vehicle, multi-view cameras, millimeter-wave radar, sensor fusion

## Abstract

Three-dimensional object detection based on the fusion of millimeter-wave radar and cameras is increasingly gaining attention due to characteristics of low cost, high accuracy, and strong robustness. Recently, the bird’s eye view (BEV) fusion paradigm has dominated radar–camera fusion-based 3D object detection methods. In the BEV fusion paradigm, the detection accuracy is jointly determined by the precision of both image BEV features and radar BEV features. The precision of image BEV features is significantly influenced by depth estimation accuracy, whereas estimating depth from a monocular image is naturally a challenging, ill-posed problem. In this article, we propose a novel approach to enhance depth estimation accuracy by fusing camera perspective view (PV) features and radar perspective view features, thereby improving the precision of image BEV features. The refined image BEV features are then fused with radar BEV features to achieve more accurate 3D object detection results. To realize PV fusion, we designed a radar image generation module based on radar cross-section (RCS) and depth information, accurately projecting radar data into the camera view to generate radar images. The radar images are used to extract radar PV features. We present a cross-modal feature fusion module using the attention mechanism to dynamically fuse radar PV features with camera PV features. Comprehensive evaluations on the nuScenes 3D object detection dataset demonstrate that the proposed dual-view fusion paradigm outperforms the BEV fusion paradigm, achieving state-of-the-art performance with 64.2 NDS and 56.3 mAP.

## 1. Introduction

Accurate and robust 3D object detection is critical for autonomous driving applications [1,2]. Autonomous vehicles are often equipped with LiDAR sensors for environmental perception. Whereas LiDAR can accurately detect the position and dimensions of objects, its high cost, inability to capture appearance information, and sensitivity to environmental conditions limit its practicality [3]. With advancements in computer vision technology, multi-view cameras have emerged as a more economical alternative for perception in autonomous vehicles. Cameras can detect rich semantic information; however, they cannot capture precise localization information. Moreover, as passive sensors, cameras are susceptible to varying lighting conditions (e.g., glare, low contrast, or low-light environments) [4]. Millimeter-wave radar can reliably detect object location without being affected by weather conditions, making it a cost-effective sensor that complements cameras well [5]. Consequently, radar–camera fusion perception has garnered increasing research interest in recent years.

Three-dimensional object detection is a critical component of perception tasks in autonomous driving. Recent advancements in radar–camera fusion-based 3D object detection predominantly rely on the bird’s eye view (BEV) fusion paradigm, where multi-view image features and radar features are transformed into the BEV space for processing [4,6,7]. The detection accuracy is jointly determined by the precision of both image BEV features and radar BEV features. The generation of image BEV features can be broadly categorized into geometry-based methods (e.g., the LSS [8] family) and learning-based methods (e.g., the BEVFormer [9] family). Due to the higher computational complexity of transformer modules compared with convolutional counterparts, geometry-based methods are more widely adopted in industrial applications and thus constitute the focus of this work. The precision of image BEV features in the LSS-based pipeline is highly dependent on the accuracy of depth estimation [10]. However, estimating depth from a monocular image is naturally a challenging, ill-posed problem. To address this problem, we aim to improve the accuracy of depth estimation with the help of radar information.

In this article, we generate image BEV features using the LSS framework. Building upon the existing BEV fusion paradigm, we innovatively incorporate the PV fusion paradigm to further enhance radar–camera fusion-based 3D object detection. Specifically, we first fuse camera PV features and radar PV features, aiming to improve the precision of image BEV features. We designed a radar image generation module utilizing radar cross-section (RCS) and depth information to accurately project radar data into the camera view. RCS information represents the object’s size [4], which determines the projection area, and depth information represents the distance, allowing us to further adjust the projection area according to the “near large, far small” imaging principle. The pixel values of the projection area are filled using radar depth information. Camera PV features and radar PV features are generated through CNNs separately. We present a cross-modal feature fusion module based on the attention mechanism to dynamically fuse camera PV features and radar PV features as radar data often suffer from sparsity, noise, and ambiguous measurements. Image BEV features are generated based on the fused PV features through view transformation. We fused the refined image BEV features with radar BEV features to produce the final 3D object detection results. Extensive experiments on the nuScenes dataset demonstrate that our method outperforms BEV fusion-based methods on 3D detection tasks.

The main contributions of this work are summarized as follows:

- We propose a novel LSS-based dual-view fusion paradigm that integrates PV fusion and BEV fusion paradigms, significantly enhancing the accuracy of 3D object detection.
- We designed a dedicated radar image generation module for PV fusion, which accurately projects radar data into the camera view by leveraging radar RCS and depth information, laying a solid foundation for extracting radar PV features.
- We introduce a cross-modal feature fusion module, employing the deformable cross-attention mechanism to efficiently and dynamically fuse PV features from different modalities, supporting the generation of more accurate image BEV features.
- The proposed method achieves state-of-the-art performance for radar–camera fusion-based 3D object detection on the nuScenes dataset.

## 2. Related Work

### 2.1. Camera-Based 3D Object Detection

Two-dimensional object detection has been extensively studied and provides the foundation for subsequent 3D perception tasks [11,12]. However, detecting 3D objects using monocular camera image presents significant challenges due to the inherent lack of depth information. To address this, FCOS3D [13] extends 2D object detection methods [14] by estimating object distances to achieve 3D object detection. DD3D [15] improves detection accuracy through pretraining on depth estimation datasets. GUPNet [16] leverages geometric constraints and shape priors to infer more reliable object depth information. MonoAMP [17] is an adaptive multi-order perceptual aggregation algorithm for enhancing the intersection of cross-dimensional feature attention.

With the release of the nuScenes dataset [3], 3D object detection using multi-view cameras has become a popular research topic. A straightforward approach involves applying monocular 3D object detection methods independently to each camera and then merging the results to form the final detection outputs. However, this method is complex and performs sub-optimally. Current approaches transform image features into the BEV space to form BEV features, which are subsequently used for 3D object detection, demonstrating promising results. These approaches can be categorized into geometry-based methods and learning-based methods.

Geometry-based methods follow the Lift–Splat–Shoot (LSS) framework [8] by explicitly estimating the depth distribution of images. This enables the transformation of 2D context features into the 3D camera frustum space, followed by sum pooling to generate BEV features. CaDDN [18] builds upon the LSS framework and achieves notable performance in 3D object detection. BEVDet [19] is a view transformer module based on LSS and a tailored non-maximum suppression strategy for 3D object detection. BEVDet4D [20] extends BEVDet by incorporating historical BEV features, effectively reducing velocity errors for 3D objects. BEVDepth [21] highlights the critical role of depth estimation in the LSS framework and proposes supervised training of the depth estimation module using LiDAR data as ground truth, significantly enhancing 3D detection accuracy. LST-BEV [22] proposes a Long-Range Cross-Task Detection Head to capture long-range dependencies and cross-task information for accurate predictions.

Learning-based methods implicitly model the transformation from a perspective view to a bird’s eye view using attention mechanisms. BEVFormer [9] constructs BEV queries and incorporates multi-scale deformable attention to locate and aggregate corresponding image features into BEV representations. PETR [23] encodes 3D positional attributes into image features, generating 3D position-aware features and avoiding the need for complex 2D-to-3D projections. Building on PETR, PETRv2 [24] introduces temporal information from prior frames to boost 3D object detection performance. Recently, the authors of [25] developed StreamPETR, an object-centric temporal mechanism to propagate long-term historical information frame by frame. SparseBEV [26] represents a method to dynamically capture BEV features and temporal information, enhancing the performance of query-based paradigms.

### 2.2. Radar–Camera 3D Object Detection

Due to the rich semantic information embedded in images, camera-based methods can distinguish objects even at long distances. However, accurately localizing objects from images remains a challenging, ill-posed problem. Moreover, cameras, as passive sensors, are highly susceptible to environmental lighting conditions. In contrast, millimeter-wave radar provides precise object localization and exhibits robust performance across various environmental conditions. Consequently, the combination of millimeter-wave radar and multi-view cameras for 3D object detection has garnered significant attention in recent years.

RadarNet [27] proposes a multi-level fusion approach that leverages both the geometric and dynamic properties of radar data to improve the accuracy of detecting distant objects and estimating their velocities. GRIFNet [28] is an explicit gating mechanism for adaptively fusing the region-of-interest (ROI) proposals generated from radar and camera data. CenterFusion [29] is a multi-stage radar–camera fusion framework, which begins by predicting initial 3D object detection boxes using image data. These boxes are then associated with radar data, and the matched radar features are used to refine the initial predictions, yielding the final 3D detection results. MVFusion [30] presents a semantic-aligned radar encoder module to align radar and camera features, alongside a radar-guided fusion transformer to enhance cross-modal correlations at a global level. SimpleBEV [31] identifies critical design and training factors for multi-sensor BEV perception systems. CRAFT [32] proposes a proposal-level early fusion approach that effectively utilizes the spatial attributes of radar and the semantic attributes of cameras. RADIANT [33] is a network that predicts 3D offsets between radar returns and object centers, leveraging radar depth information to enhance 3D detection accuracy.

More recently, the dominant paradigm for radar–camera fusion has been to transform radar and image features into the BEV space for 3D object detection. According to the different strategies for generating image BEV features, existing methods can be broadly categorized into LSS-based fusion schemes and transformer-based fusion schemes. In terms of LSS-based approaches, CRN [7] utilizes radar occupancy information to improve the accuracy of converting image features into BEV space and employs the attention mechanism to fuse BEV features from both modalities. RCBEVDet [4] introduces a RadarBEVNet, consisting of a dual-stream radar backbone and an RCS-aware BEV encoder, to better extract radar BEV features. Regarding transformer-based approaches, RCBEVDet++ [34], an extended version of RCBEVDet, replaces the LSS-based image BEV generation module with a transformer-based design, further boosting 3D object detection performance. DPFT [35] exploits low-level radar representations (RA and AE maps) and samples query points in 3D space to fuse image and radar features, achieving strong robustness under adverse weather conditions. In this work, we focus on improving LSS-based fusion schemes owing to their simplicity, efficiency, and widespread adoption in industrial applications. To this end, we propose a dual-view fusion paradigm, which innovatively integrates the PV fusion paradigm into the BEV fusion framework. By enhancing the precision of image BEV features, our approach significantly improves the performance of 3D object detection.

## 3. Method

### 3.1. Preliminary

LSS [8] is the pioneering work that generates BEV scene representations from multi-view RGB images. The input consists of *n* RGB images ${\left\{X_{k}\in R^{3\times H\times W}\right\}}_{n}$, where *H* and *W* denote the height and width of each image, respectively. Each image is associated with an extrinsic matrix $E_{k}\in R^{3\times 4}$ and an intrinsic matrix $I_{k}\in R^{3\times 3}$. LSS then seeks to generate a rasterized representation of the scene in the BEV coordinate frame, denoted as $F^{bev}\in R^{C\times X\times Y}$, where X×Y defines the spatial extent of the horizontal plane in the physical world, and *C* represents the feature dimension at each spatial location.

The core operations of LSS consist of “lift” and “splat”. The “lift” operation aims to recover the depth of each pixel in the image, thereby projecting the image from the 2D plane into the 3D space. This process is divided into two steps: The first step is the generation of the 3D frustum point cloud. Given an image of size (H,W), each pixel is associated with *D* discrete depth values, representing all possible depth positions that the pixel may occupy. This process produces a frustum point cloud of size (D,H,W). The second step is the generation of the context feature point cloud. A convolutional neural network is employed as the backbone to extract image features. For each point on the feature map, a *C*-dimensional feature vector and a probability distribution over *D* discrete depth values are predicted. The outer product of the feature vector and the depth distribution is then computed, resulting in the construction of the context feature point cloud.

The “splat” operation refers to the projection of the context features onto the BEV grid for the construction of BEV representations. The procedure is as follows: first, by leveraging both the intrinsic and extrinsic matrices of the camera, the entire frustum point cloud is transformed into the ego-vehicle coordinate system. Second, the frustum point cloud is translated from the ego-vehicle coordinate system into the BEV grid, whereas points that fall outside the grid boundaries are discarded. Finally, the context features associated with points residing in the same grid cell are aggregated through sum pooling, thereby yielding the final BEV features.

### 3.2. Motivation

The BEV space is particularly suitable for 3D object detection tasks as transforming features into the BEV space enables better capture of key attributes of 3D objects, such as position, scale, and orientation [19]. In radar–camera BEV fusion, the typical process involves independently extracting radar BEV features and image BEV features, which are then fused and processed through a 3D detection head to output the final predictions. Since radar inherently provides accurate spatial information, the extracted radar BEV features are reliable. However, extracting accurate image BEV features remains challenging due to the difficulty of estimating precise depth information from monocular images. The LSS method implicitly learns depth estimation through supervision from the final detection loss. BEVDepth highlights that the LSS approach yields suboptimal depth quality and addresses this problem by directly supervising the depth estimation with LiDAR ground truth. This modification in training strategy significantly enhances detection accuracy [21]. In this work, we investigate the integration of radar information into the LSS pipeline to further enhance the accuracy of 3D object detection.

### 3.3. Overall Architecture

**Figure 1:**
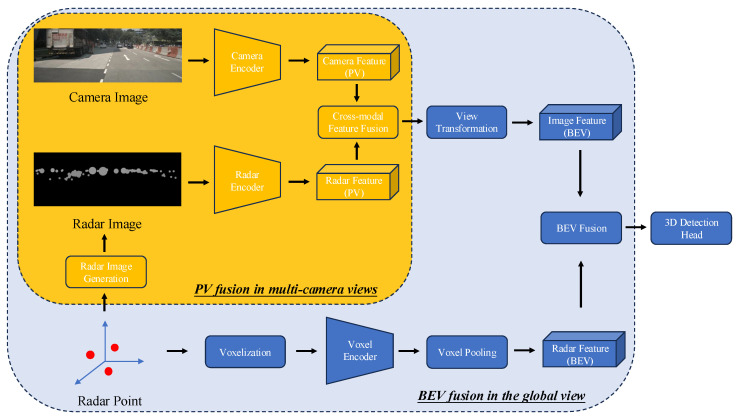
The overall framework of the proposed dual-view fusion paradigm. Radar points are encoded and transformed into the bird’s eye view to generate the radar BEV features. Concurrently, radar points are sent to the proposed radar image generation module to generate radar images. Afterward, camera images and radar images are processed by modality-specific encoders to extract PV features. The cross-modal feature fusion module is then utilized to integrate the PV features from both modalities, producing fused PV features. The fused PV features is employed to generate the image BEV features. Finally, radar BEV features and image BEV features are fused and processed through a 3D detection head to output 3D detection results.

This study proposes a radar–camera fusion framework that integrates PV fusion and BEV fusion to enhance 3D object detection performance for autonomous vehicles. An overview of the proposed framework is depicted in Figure 1. The process consists of extracting radar BEV features and image BEV features, which are then fused and processed through a 3D detection head to output detection results. Extracting radar BEV features involves voxelizing the radar point cloud, followed by voxel encoding that extracts radar voxel features. The voxel pooling operation is then used to convert voxel features into BEV features. The extraction of image BEV features follows the Lift–Splat–Shoot pipeline. First, we generate radar images of multi-camera views using the radar image generation module. Subsequently, CNNs are employed to extract camera PV features and radar PV features separately. The cross-modal feature fusion module is then utilized to integrate the PV features from both modalities, producing fused PV features. The view transformation module uses the fused PV features to extract semantic features and predict the depth distribution. The predicted depth distribution is leveraged to generate a frustum point cloud, where the extracted semantic features are assigned to the points. Finally, the splat operation converts the point cloud features into BEV features. The following sections provide a detailed explanation of PV fusion and BEV fusion.

### 3.4. PV Fusion

First, we generate radar images for each camera view using the radar image generation module. Subsequently, the camera encoder and radar encoder are applied to extract the respective PV features. The camera encoder utilizes a suitable backbone network for visual tasks (e.g., ResNet [36]) and a neck model (e.g., FPN [37]) to extract 16× downsampled image feature maps (i.e., camera PV features). The radar encoder is designed based on ResNet and consists of two main components: the stem and the block. The stem is the original stem module of ResNet and is responsible for processing the input data. The block follows the architecture of the first stage of ResNet50, utilizing two residual blocks to generate 16× downsampled radar feature maps (i.e., radar PV features). Finally, the cross-modal feature fusion module fuses the PV features extracted from both modalities. This fusion process enables the integration of complementary information from the camera and radar data. Next, we provide a detailed description of the radar image generation module and the cross-modal feature fusion module.

**Figure 2:**
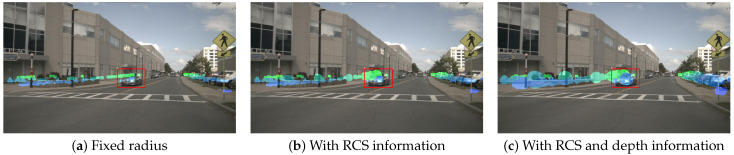
Comparison of different radar image generation methods. (**a**) The radar projection area with a fixed radius does not account for object size and distance. (**b**) The radar projection area with RCS information considers object size but neglects the imaging principle of “near large, far small”. (**c**) The radar projection area with both RCS and depth information takes into account both object size and distance, resulting in a more accurate projection area. (Green indicates farther distances, whereas blue represents closer distances. Best viewed in color.)

**Radar Image Generation.** The radar processes scan data to detect and identify targets, yielding a set of identified objects. Each identified target includes measurements such as the position, velocity, and radar cross-section. Using the radar’s position information, we project the radar data into the camera view. The projected image locations $Loc_{img}\in R^{3\times 1}$ are computed as follow:

(1) $$Loc_{img}=I_{k}E_{k}Loc_{radar}$$

where $I_{k}$ represents the camera’s intrinsic parameter matrix, $E_{k}$ is the extrinsic calibration matrix from radar to camera, and $Loc_{radar}\in R^{4\times 1}$ denotes the target’s location in the radar coordinate system. Both $Loc_{img}$ and $Loc_{radar}$ are represented in homogeneous coordinates. To mitigate the influence of radar measurement uncertainty, previous works [38,39] marked each target’s position in the image as a small circle rather than a single pixel, as shown in Figure 2a. The pixels inside the circle are filled with the radar’s depth or velocity information, whereas other areas are filled with zeros. Additionally, for overlapping circles, only the information of the closer target is retained. The image generated through this process is referred to as the radar image.

Previous works set an empirically determined, fixed circle radius *r* to define the projected area of radar data. However, different targets vary in size and distance, making such a fixed projection area inherently inaccurate and potentially misleading the extraction of radar PV features. As shown in the red box of Figure 2a, the radar information corresponding to the building in the background is incorrectly projected onto the car in the foreground.

In this work, we propose a radar image generation module based on radar RCS and depth information, aiming to enhance the accuracy of radar projection area in the camera view. Specifically, radar RCS information provides size-related characteristics of the target. A larger target leads to a larger RCS measurement [4]. Therefore, we dynamically adjust the projection area of each radar target based on the RCS information. The circle radius *r* is scaled by an RCS modulation factor $R_{rcs}$, as described by the following equations:

(2) $$R_{rcs}=max\left({\bar{V}}_{rcs},0\right)+1$$

(3) $${\bar{V}}_{rcs}=\frac{V_{rcs}-V_{minrcs}}{V_{maxrcs}-V_{minrcs}}$$

where $V_{rcs}$ denotes the RCS value of the radar target, measured in square meters ($m^{2}$). $V_{maxrcs}$ and $V_{minrcs}$ represent the maximum and minimum RCS values of the radar, respectively, and ${\bar{V}}_{rcs}$ indicates the normalized RCS value of the radar target. The radar image adjusted based on the RCS information is shown in Figure 2b, where the radar projection area of the building in the background is noticeably expanded, covering a larger portion of the target. However, this also increases the erroneous projection onto the car in the foreground. This occurs because the imaging of targets must adhere to the rule of “near large, far small”. Even if a target is large, its projected area in the camera view will be smaller if it is farther away. Therefore, we further dynamically adjust the projection area of each radar target based on depth information. The circle radius *r* applies an additional depth modulation factor $R_{depth}$, as described by the following equations:

(4) $$R_{depth}=\frac{e^{\alpha }-e^{-\alpha }}{e^{\alpha }+e^{-\alpha }}+1$$

(5) $$\alpha =\frac{DEPTH_{max}}{V_{depth}}-1$$

where $V_{depth}$ represents the radar target depth value, measured in meters (m). $DEPTH_{max}$ refers to the maximum depth value in the scene. The radar image adjusted based on both RCS and depth information is shown in Figure 2c, where the radar projection areas for both the car in the foreground and the building in the background are more accurate. According to the experiments presented in Section 4, the radar image generation method based on RCS and depth information attains 57.2 NDS and 48.4 mAP, demonstrating superior performance compared to the other approaches.

**Cross-modal Feature Fusion.** Whereas radar provides a wealth of useful information, it also presents several challenges, such as noisy measurements induced by multi-path effects or clutter [7]. Through the radar image generation module, all radar targets, including noisy targets, are projected into the camera view. The radar PV features extracted from this radar image are inherently noisy. Applying naive fusion methods, such as channel-wise concatenation or summation, does not resolve this problem and may introduce adverse effects. In this work, we propose a dynamic fusion approach using the attention mechanism [40] to fuse camera PV features with radar PV features, achieving promising results.

Specifically, given the camera PV features denoted by $F_{c}^{pv}\in R^{C\times H\times W}$ and the radar PV features denoted by $F_{r}^{pv}\in R^{C\times H\times W}$, we first leverage the accurate camera PV features to update the noisy radar PV features. Specifically, $F_{c}^{pv}$ is converted into queries $z_{q_{c}}$, and $F_{r}^{pv}$ is treated as keys and values. Then we apply deformable cross-attention [41] to update the radar PV feature, as shown in the following equation:

(6) $$F_{r}^{pv}\leftarrow \sum_{m=1}^{M}W_{m}\left[\sum_{k=1}^{K}A_{mqk}\cdot \overset{´}{W_{m}}F_{r}^{pv}\left(p_{q_{c}}+\Delta p_{mqk}\right)\right]$$

where *m* indexes the attention head, *q* indexes the query element, *k* indexes the sampled keys, *M* is the total number of attention heads, and *K* is the total number of sampled keys. $p_{q_{c}}\in R^{2}$ represents the 2D reference point. $\Delta p_{mqk}$ and $A_{mqk}$ denote the sampling offset and attention weight of the $k^{th}$ sampling point of the $q^{th}$ query element in the $m^{th}$ attention head, respectively. The scalar attention weight $A_{mqk}$ is normalized in the range [0,1]. $\Delta p_{mqk}\in R^{2}$ are of 2D real numbers with unconstrained range. Both $\Delta p_{mqk}$ and $A_{mqk}$ are obtained via linear projection over the query $z_{q_{c}}$. $W_{m}\in R^{C\times C_{v}}$ and $\overset{´}{W_{m}}\in R^{C_{v}\times C}$ are the output projection matrix and input value projection matrix ($C_{v}=C/M$ by default), which are trainable on samples. Once the radar PV features $F_{r}^{pv}$ are updated, we then use them as queries $z_{q_{r}}$ and treat $F_{c}^{pv}$ as keys and values. Similarly, we apply deformable cross-attention to update the camera PV features, as shown in the following equation:

(7) $$F_{c}^{pv}\leftarrow \sum_{m=1}^{M}W_{m}\left[\sum_{k=1}^{K}A_{mqk}\cdot \overset{´}{W_{m}}F_{c}^{pv}\left(p_{q_{r}}+\Delta p_{mqk}\right)\right]$$

After updating the PV features from both modalities, they are concatenated and processed through the residual block, obtaining the final fused PV features $F_{fuse}^{pv}\in R^{C\times H\times W}$. As demonstrated in Section 4, our attention-based method achieves 57.2 NDS and 48.4 mAP, which is nearly 1.0 higher than that of other naive methods.

### 3.5. BEV Fusion

We adopt the conventional BEV feature generation and BEV feature fusion methods to obtain the fused BEV features, and finally, the 3D object detection results are obtained through the detection head. The detection head is based on CenterPoint [42], which predicts the center heatmap using an anchor-free and multi-group head [43]. Next, we introduce the components of the BEV fusion module.

**Image BEV Feature Generation.** We generate image BEV features based on the LSS framework. For each camera view, we first perform PV fusion to obtain the fused PV features $F_{fuse}^{pv}\in R^{H\times W}$ (ignoring the channel dimension). Then, based on the fused PV features, we predict the depth distribution $d\in R^{D}$ and semantic features $c\in R^{C}$ for each pixel and compute the outer product to obtain the frustum view features $F_{fuse}^{fv}\in R^{H\times W\times D\times C}$. After completing the above processing, we use the splat operation to convert the frustum view features $F_{fuse}^{fv}$ into the unified BEV features $F_{img}^{bev}\in R^{X\times Y\times C}$. For further details, we refer the reader to LSS [8].

**Radar BEV Feature Generation.** We generate radar BEV features based on the PointPillars framework. First, we voxelize the radar point cloud in the frustum view $V_{radar}^{fv}\in R^{X\times Y\times Z}$ (ignoring the feature dimension), where Z=1 denotes the pillar-style voxelization. Next, we use PointNet [44] and sparse convolution [45] to encode the non-empty radar pillars into frustum view features $F_{radar}^{fv}\in R^{X\times Y\times Z\times C}$. Finally, we apply the pooling operation [46] to convert the frustum view features $F_{radar}^{fv}$ into the unified BEV features $F_{radar}^{bev}\in R^{X\times Y\times C}$. For further details, we refer the reader to PointPillars [47].

**BEV Feature Fusion.** We fuse the image BEV features and radar BEV features based on the CRN framework. First, the image BEV features and radar BEV features are flattened, after which each feature is passed through a layer normalization layer. Then, the features are concatenated and transformed into a *C*-dimensional query feature via a linear projection layer. Finally, the feature map is aggregated through the multi-modal deformable cross attention (MDCA) module. We refer the reader to CRN [7] for more details.

## 4. Experiments

### 4.1. Experimental Settings

**Dataset and Metrics.** We conducted our experiments on the popular large-scale autonomous driving dataset, nuScenes [3], which comprises 1000 diverse and complex driving scenarios collected in Boston and Singapore. Among these, 700 scenes are designated for training, 150 for validation, and the remaining 150 for testing. The dataset is equipped with 6 RGB cameras, 5 mmWave radar sensors, and 1 LiDAR sensor, providing 3D annotations at a frequency of 2 Hz. nuScenes offers a comprehensive set of metrics to evaluate the performance of 3D object detection algorithms. First, the Average Precision (AP) metric was evaluated using the mean Average Precision (mAP), which was calculated to measure the precision and recall of detection methods. However, in nuScenes metrics, it is not defined based on the Intersection over Union (IOU) but the match by the 2D center distance on the ground plane. Second, True Positive (TP) metrics were used to evaluate the multi-aspect precision, including Average Translation Error (ATE), Average Scale Error (ASE), Average Orientation Error (AOE), Average Velocity Error (AVE), and Average Attribute Error (AAE) of the detection results. Third, the nuScenes Detection Score (NDS) was utilized to indicate the all-sided detection performance, which considers mAP and the regression quality in terms of box location, size, orientation, attributes, and velocity. As in the following equations, the above metrics were calculated over the distance matching threshold of D={0.5,1,2,4} and the set of ten classes C [48].

(8) $$mAP=\frac{1}{\left|C||D\right|}\sum_{c\in C}\sum_{d\in D}AP_{c,d}$$

(9) $$mTP=\frac{1}{\left|C\right|}\sum_{c\in C}TP_{c}$$

(10) $$NDS=\frac{1}{10}\left[5mAP+\sum_{mTP\in TP}\left(1-min\left(1,mTP\right)\right)\right]$$

**Implementation Details.** For radar image generation, depth information was utilized to fill the pixel values of the projected area in the camera view. The circle radius *r* was configured as 5 and subsequently scaled according to RCS and depth information. Regarding camera and radar encoders, the backbone produces four levels of feature maps with strides of 4, 8, 16, and 32. An FPN was further adopted to aggregate multi-scale features, with the output feature map at stride 16 concatenated. For cross-modal feature fusion, we employed 4 attention heads with 4 sampling points. The perception range was set to [−51.2,51.2] m in both the X- and Y-axis centered around the ego vehicle. We used uniform discretization with a depth range of [2.0,58.0] m and bin size of 0.5 m, resulting in D=112. The feature dimension *C* was configured as 256. The entire model was trained for 24 epochs in an end-to-end manner using the AdamW [49] optimizer. The learning rate was initialized to 2 × 10^−4^, the weight decay was set to 1 × 10^−4^, the learning rate schedule followed the step decay policy, and gradient clipping was applied with a threshold of 5. To prevent overfitting, we incorporated both image- and BEV-level data augmentation strategies [21]. For image-level augmentation, we applied resizing, cropping, and horizontal flipping. For BEV-level augmentation, we utilized random flipping along the X- and Y-axis, global rotation within the range of [−π/8,π/8], and global scaling between [0.95,1.05]. During training, radar sweeps and points were randomly dropped [50] to further improve robustness. All training and inference experiments were conducted on a workstation equipped with an Intel Core i9 CPU and RTX 3090 GPUs. Other implementation and training procedures followed standard practices as described in [7].

### 4.2. Comparison with State of the Art

**Figure 3:**
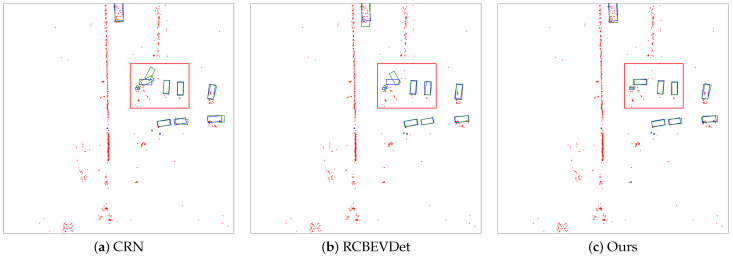
Qualitative results from CRN [7], RCBEVDet [4], and our method. Green boxes indicate prediction boxes, blue boxes represent ground truth boxes, and red dots are radar points. Best viewed in color.

We compare the proposed method with previous state-of-the-art 3D detection methods on the nuScenes val and test sets in Table 1 and Table 2, respectively. As shown in Table 1, our method achieves state-of-the-art results within LSS-based fusion schemes and further surpasses the results of several transformer-based fusion schemes. Our method ranks first across different input image sizes and backbone settings in terms of the NDS metric. Compared to the CRN method [7], our approach improves by 1.4 in NDS and 0.9 in mAP. Compared with the RCBEVDet method [4], our method shows an improvement of 0.4 in NDS and 2.9 in mAP. These performance improvements over the BEV fusion paradigm demonstrate the effectiveness of the dual-view fusion paradigm. Qualitative comparisons are presented in Figure 3. As highlighted by the targets within the red bounding boxes, our method demonstrates superior detection capabilities, accurately identifying object’s position, scale, and orientation. Furthermore, compared to the camera-only state-of-the-art method, BEVDepth [21], our method shows an improvement of 7.1 in NDS and 12.2 in mAP, highlighting the effectiveness of integrating radar information in comparison to camera-only methods. Our method also outperforms the LiDAR-based method CenterPoint [42], proving the potential for autonomous vehicles to utilize cost-effective cameras and radar to replace LiDAR for 3D environmental perception.

**Table 1:** Comparison of 3D object detection results on nuScenes val set. “L”, “C”, and “R” represent LiDAR, camera, and radar, respectively. *: BEV fusion paradigm. ^†^: trained with CBGS.

Method	Input	Backbone	Image Size	NDS↑	mAP↑
CenterPoint [42]	L	-	-	59.8	49.4
CRN * [7]	C + R	R18	256×704	54.3	44.8
RCBEVDet * ^†^ [4]	C + R	R18	256×704	54.8	42.9
Ours	C + R	R18	256×704	**55.2**	**45.6**
BEVDet ^†^ [19]	C	R50	256×704	39.2	31.2
BEVDepth ^†^ [21]	C	R50	256×704	47.5	35.1
SOLOFusion ^†^ [51]	C	R50	256×704	53.4	42.7
StreamPETR [25]	C	R50	256×704	54.0	43.2
CRN * [7]	C + R	R50	256×704	56.0	**49.0**
RCBEVDet * ^†^ [4]	C + R	R50	256×704	56.8	45.3
Ours	C + R	R50	256×704	**57.2**	48.4
BEVDepth ^†^ [21]	C	R101	512×1408	53.5	41.2
SOLOFusion ^†^ [51]	C	R101	512×1408	58.2	48.3
StreamPETR [25]	C	R101	512×1408	59.2	50.4
CRN * [7]	C + R	R101	512×1408	59.2	52.5
Ours	C + R	R101	512×1408	**60.6**	**53.4**

Note: Bold indicates the best result.

**Table 2:** Comparison of 3D object detection results on nuScenes test set. “L”, “C”, and “R” represent LiDAR, camera, and radar, respectively. *: BEV fusion paradigm.

Method	Input	Backbone	NDS↑	mAP↑
PointPillars [47]	L	Pillars	55.0	40.1
KPConvPillars [52]	R	Pillars	13.9	4.9
CenterFusion [29]	C + R	DLA34	44.9	32.6
RCBEV * [48]	C + R	Swin-T	48.6	40.6
MVFusion [30]	C + R	V2-99	51.7	45.3
CRAFT [32]	C + R	DLA34	52.3	41.1
BEVFormer [9]	C	V2-99	56.9	48.1
PETRv2 [24]	C	V2-99	58.2	49.0
BEVDepth [21]	C	V2-99	60.5	51.5
BEVDepth [21]	C	ConvNeXt-B	60.9	52.0
BEVStereo [46]	C	V2-99	61.0	52.5
SOLOFusion [51]	C	ConvNeXt-B	61.9	54.0
CRN * [7]	C + R	ConvNeXt-B	62.4	**57.5**
SparseBEV [26]	C	V2-99	63.6	55.6
StreamPETR [25]	C	V2-99	63.6	55.0
RCBEVDet * [4]	C + R	V2-99	63.9	55.0
Ours	C + R	V2-99	**64.2**	56.3

Note: Bold indicates the best result.

As shown in Table 2, our method achieves 64.2 NDS and 56.3 mAP on the test set. Compared to the CRN method using the ConvNeXt-Base backbone, our approach utilizes the smaller V2-99 backbone but achieves a higher NDS score (+1.8 NDS). Furthermore, our method significantly outperforms the RCBEVDet method in both the NDS and mAP metrics using the same backbone. Specifically, the NDS score is improved by 0.4, and the mAP score is improved by 1.3.

### 4.3. Ablation Studies

We conducted ablation studies on nuScenes val set to analyze the effectiveness of each proposed module in PV fusion. We adopt R50 backbone, 256×704 image size, and 128×128 BEV size as the model setting.

**PV fusion.** Our method improves upon CRN (BEV fusion paradigm) by integrating it with the PV fusion described in Section 3. CRN utilizes the RVT module to enhance the precision of image BEV features, whereas our approach enhances image BEV features through PV fusion. To evaluate the effectiveness of the proposed modules, we incrementally removed and added modules, transitioning from the CRN method to our method. As shown in Table 3, we first removed the RVT module from CRN, resulting in a decrease of 0.4 in NDS and 0.9 in mAP. Next, we introduced PV fusion, starting with the radar image generation module to generate radar images and then using the cross-modal feature fusion module as the fusion method. This configuration achieves 57.2 in NDS and 48.4 in mAP, demonstrating the effectiveness of PV fusion and validating the superiority of the dual-view fusion paradigm.

**Radar Image Generation.** We conducted ablation experiments for the design of the radar image generation module, as shown in Table 4. Traditional methods employ a fixed circle radius to determine the projection area of radar data, achieving 56.6 NDS and 48.0 mAP. By incorporating target size (RCS) information to dynamically adjust the projection area, we observe no significant improvement in the NDS metric and a slight decrease in the mAP metric. We argue that this is due to the failure to account for the “near large, far small” principle of camera imaging. Therefore, we further integrated depth information of the target, ultimately achieving results of 57.2 NDS and 48.4 mAP, demonstrating the effectiveness of the proposed radar image generation module.

**Cross-modal Feature Fusion.** We conducted ablation experiments to evaluate the design of the cross-modal feature fusion module, as shown in Table 5. We compare four different fusion methods: Multiply Fusion [39], Add Fusion [53], Concatenation Fusion [54], and Attention Fusion. Our proposed Attention Fusion outperforms the other fusion methods, achieving an improvement of approximately 1.0 in both the NDS and mAP. We argue that radar PV features are noisy. Multiply Fusion, Add Fusion, and Concatenation Fusion are unable to distinguish noisy features, which leads to degraded fusion performance. In contrast, by using Attention Fusion, we leverage attention weights to dynamically fuse features, reducing the interference from noisy features and effectively improving the final fusion result.

### 4.4. Analysis

We analyzed the effectiveness of the proposed approach and conducted a comparative study against two closely related state-of-the-art methods, CRN [7] and RCBEVDet [4]. To this end, we provide a concise comparison table that contrasts these methods in terms of depth estimation methodology, radar feature processing strategy, and fusion stage. As shown in Table 6, our method differs in several key aspects. First, whereas CRN and RCBEVDet estimate depth solely from camera information, our approach leverages both radar and camera data for depth estimation. Second, both our method and CRN exploit radar information during the generation of image BEV features and radar BEV features, whereas RCBEVDet employs radar information exclusively for generating radar BEV features. Finally, unlike CRN and RCBEVDet, which perform feature fusion only in the BEV space, our approach conducts feature fusion twice, in both the PV space and the BEV space, thereby enabling more effective cross-modal interaction.

**Figure 4:**
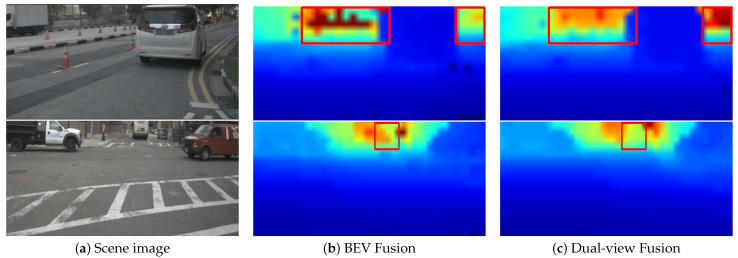
Comparison of depth map. From the figure, we can see that the estimation of dual-view fusion is of better quality than that of BEV fusion. The red boxes highlight the regions where the BEV fusion exhibits significant errors, whereas dual-view fusion provides accurate predictions.

**Depth Estimation.** In the BEV fusion paradigm, depth estimation is supervised by LiDAR ground truth. In the dual-view fusion paradigm, we introduced PV fusion, aiming to further enhance depth estimation accuracy by integrating radar depth information. To analyze the quality of learned depth in both paradigms, we evaluated the learned depth on the nuScenes val set using commonly adopted depth estimation metrics, including scale-invariant logarithmic error (SILog), mean absolute relative error (AbsRel), mean squared relative error (SqRel), and root mean squared error (RMSE). The results are presented in Table 7. The depth estimation quality of the dual-view fusion paradigm is significantly higher that of the BEV fusion paradigm. For instance, the BEV fusion paradigm only achieves an AbsRel of 3.88, whereas the dual-view fusion paradigm significantly reduces it to 0.14 AbsRel. Furthermore, we compare the depth maps estimated by both methods. As illustrated in Figure 4, the depth map predicted by the dual-view fusion paradigm is more accurate than that generated by the BEV fusion paradigm. Specifically, in the scene highlighted by the red box, the BEV fusion paradigm produces an evidently incorrect depth prediction, whereas the dual-view fusion paradigm provides a precise estimate. These results collectively demonstrate the effectiveness of the dual-view fusion paradigm in improving depth estimation accuracy.

**Image BEV Features.** We analyzed the precision of image BEV features generated by different methods. To this end, we directly generated 3D detection results from the image BEV features, as presented in Table 8. Using BEVDepth as the baseline, we incorporated the RVT module proposed in CRN [7] and the PV fusion module introduced in this work. Both modules aim to enhance the precision of image BEV features by leveraging radar information. It can be observed that our proposed method achieves superior performance. Compared to the RVT module, the PV fusion module further improves NDS by 0.9 and mAP by 1.4. This demonstrates that the image BEV features generated by our method are more accurate, validating the effectiveness of our proposed PV fusion approach.

**Robustness.** To systematically analyze the robustness of different methods, we dropped either image or radar inputs during the model inference stage and evaluated the mAP metric, as shown in Table 9. Our method consistently performs the best under various sensor failure scenarios, maintaining the highest mAP scores. Additionally, we observe that as the number of valid radar or image inputs increases, the performance of our method improves more significantly. When radar or image sensors all fail, our method shows a modest improvement of 0.2 over CRN. However, when both radar and image sensors are fully valid, our method achieves a notable improvement of 1.2 over CRN. This further highlights the superior capability of our method in utilizing both image and radar data.

**Weather and Lighting.** We analyzed the algorithm’s performance under different weather and lighting conditions, as shown in Table 10. The radar–camera algorithm shows a significant improvement over the camera-only algorithm. Our method achieves more than 10 mAP improvement compared to BEVDepth [21] under all conditions. Compared to state-of-the-art radar–camera fusion algorithms, our method also performs the best. Specifically, in sunny conditions, we observe a 1.3 mAP improvement; in rainy conditions, a 0.6 mAP improvement; a 1.1 mAP improvement during the day; and a 1.5 mAP improvement at night. This consistent improvement demonstrates that our method is better at integrating radar information and leveraging radar’s robustness to enhance 3D object detection accuracy across various environmental conditions.

**Computational Efficiency.** The performance across diverse configurations has been validated in the aforementioned paragraph, and resource consumption during model deployment will be further analyzed. We provide details on parameters, GFLOPs, and FPS for our method and rival ones, as shown in Table 11. All methods were evaluated with the R18 backbone and 256×704 image size, and detection metrics are presented in Table 1. Thanks to the proposed PV fusion, which enables more effective integration of radar and image information, our approach only requires lightweight feature extraction modules to achieve superior performance. Specifically, our method requires just 130.4 GFLOPs of computational budget and can be processed at 30.1 FPS.

## 5. Discussion

In this study, we propose a novel dual-view radar–camera fusion paradigm for 3D object detection. Compared with existing BEV fusion methods, our approach innovatively incorporates PV fusion, introducing radar depth information into the process of generating image BEV features, thereby improving 3D object detection performance. The key advantage of our method lies in its more effective radar–camera fusion strategy, which achieves superior detection accuracy with a relatively lightweight model design.

Our work clearly identifies the bottleneck in the generation of image BEV features, which is the accuracy of the depth estimation module. Estimating scene depth from a monocular image is an inherently ill-posed problem. However, radar naturally provides precise depth measurements. By effectively integrating this information into the depth estimation module, we significantly enhance its accuracy (see Table 7). As the depth estimation improves, the accuracy of the image BEV features is also substantially boosted. Directly applying these refined image BEV features for 3D object detection already yields superior performance compared with existing approaches (see Table 8). Finally, by further fusing image BEV features with radar BEV features, our method achieves state-of-the-art tested results.

Nevertheless, our approach also has limitations. In particular, it relies on high-quality sensor calibration, since PV fusion requires projecting radar data into the image coordinate system using calibration matrices to extract radar PV features. Misalignment between radar and the camera could potentially degrade performance [55]. In future work, we plan to incorporate attention mechanisms to learn adaptive cross-modal alignment, thereby reducing the reliance on precise calibration.

The proposed dual-view fusion paradigm demonstrates the effectiveness of incorporating radar information into the process of generating image BEV features, enabling advanced 3D object detection. Looking ahead, we will also explore alternative radar–camera fusion strategies, such as integrating image cues into the generation of radar BEV features, with the goal of further improving detection accuracy. With continuous advances in radar–camera fusion, this low-cost sensor solution has the potential to replace expensive LiDAR-based systems, providing reliable and robust environmental perception for autonomous driving vehicles.

In summary, this study not only advances the state of the art in radar–camera fusion for 3D object detection but also lays a solid foundation for practical, scalable, and cost-effective perception solutions in autonomous driving.

## 6. Conclusions

In this study, we present the dual-view fusion paradigm, a novel radar–camera fusion framework for 3D object detection. The paradigm incorporates the PV fusion strategy into the BEV fusion pipeline, where PV fusion enhances the accuracy of depth estimation and thereby refines the quality of image BEV features. These refined image BEV features are subsequently fused with radar BEV features, leading to more precise and reliable 3D detection results. To achieve this, we developed a radar image generation module based on RCS and depth cues, enabling accurate radar-to-camera projection, and introduced a cross-modal feature fusion module that employs an attention mechanism to dynamically integrate complementary features from radar and camera modalities.

Extensive experiments validate the effectiveness of our design, demonstrating that the proposed dual-view fusion paradigm consistently outperforms the conventional BEV fusion approach and achieves state-of-the-art detection accuracy. These results highlight the potential of dual-view fusion as a general framework for radar–camera perception and indicate promising directions for future research, such as extending the paradigm to other multimodal tasks and exploring more efficient fusion strategies for embedded deployment.

## Figures and Tables

**Table 3:** Ablation of PV fusion.

Method	NDS↑	mAP↑
CRN [7]	55.7	47.4
−RVT	55.3↓0.4	46.5↓0.9
+PV fusion	**57.2** ↑1.9	**48.4** ↑1.9

Note: Red upward arrows indicate improvement, blue downward arrows indicate decrease, and bold numbers denote the best results.

**Table 4:** Ablation of radar image generation methods.

Method	NDS↑	mAP↑
Fixed Radius	56.6	48.0
+RCS	56.7↑0.1	47.7↓0.3
+Depth	**57.2** ↑0.6	**48.4** ↑0.4

Note: Red upward arrows indicate improvement, blue downward arrows indicate decrease, and bold numbers denote the best results.

**Table 5:** Ablation of cross-modal feature fusion methods.

Method	NDS↑	mAP↑
Multiply Fusion	56.2	47.3
Add Fusion	56.3	47.2
Concat Fusion	56.2	47.5
Attention Fusion	**57.2**	**48.4**

Note: Bold indicates the best result.

**Table 6:** Comparison of different methods in terms of depth estimation, radar processing, and fusion stage.

Method	Depth Estimation	Radar Processing	Fusion Stage
CRN [7]	Camera	Image and Radar BEV feature	BEV fusion
RCBEVDet [4]	Camera	Radar BEV feature	BEV fusion
Ours	Camera and Radar	Image and Radar BEV feature	PV and BEV fusion

**Table 7:** Analysis of depth estimation.

Method	SILog↓	AbsRel↓	SqRel↓	RMSE↓
BEV Fusion	8.08	3.88	53.94	27.31
Dual-view Fusion	**3.09**	**0.14**	**0.18**	**5.79**

Note: Bold indicates the best result.

**Table 8:** Analysis of image BEV features. “C” and “R” represent camera and radar, respectively.

Method	Input	NDS↑	mAP↑
BEVDepth [21]	C	47.6	37.0
+RVT [7]	C + R	54.7	45.8
+PV Fusion	C + R	**55.6**	**47.2**

Note: Bold indicates the best result.

**Table 9:** Analysis of robustness using mAP metric.

Method	Input	Drop	# of View Drops			
			0	1	3	6
BEVDepth [21]	C	C	36.72	31.96	15.18	0.00
CRN [7]	C + R	C	47.25	42.44	20.36	0.23
		R		46.15	39.54	33.56
RCBEVDet [4]	C + R	C	45.23	40.54	19.21	0.10
		R		42.95	37.88	31.17
Ours	C + R	C	**48.39**	**43.18**	**20.62**	**0.33**
		R		**47.10**	**40.06**	**33.92**

Note: Bold indicates the best result.

**Table 10:** Analysis of different lighting and weather conditions using mAP metric.

Method	Input	Sunny	Rainy	Day	Night
BEVDepth [21]	C	36.6	38.3	37.2	16.8
CRN [7]	C + R	46.8	49.6	47.6	27.6
RCBEVDet [4]	C + R	44.7	48.5	45.7	25.3
Ours	C + R	**48.1**	**50.2**	**48.7**	**29.1**

Note: Bold indicates the best result.

**Table 11:** Analysis of computational efficiency.

Method	#param.	GFLOPs	FPS
CRN [7]	37.2 M	149.1	27.9
RCBEVDet [4]	35.3 M	148.1	28.3
Ours	**31.8** M	**130.4**	**30.1**

Note: Bold indicates the best result.

## Data Availability

The data are available online at https://www.nuscenes.org/nuscenes (accessed on 27 September 2025).

## Abbreviations

The following abbreviations are used in this manuscript:

BEV	Bird’s Eye View
PV	Perspective View
RCS	Radar Cross Section
CNN	Convolutional Neural Network
LSS	Lift–Splat–Shoot
ROI	Region of Interest
FPN	Feature Pyramid Network
mAP	Mean Average Precision
IOU	Intersection over Union
TP	True Positive
ATE	Average Translation Error
ASE	Average Scale Error
AOE	Average Orientation Error
AVE	Average Velocity Error
AAE	Average Attribute Error
NDS	nuScenes Detection Score
SILog	Scale-Invariant Logarithmic Error
AbsRel	Mean Absolute Relative Error
SqRel	Mean Squared Relative Error
RMSE	Root Mean Squared Error
